# Making argumentative work visible in GenAI-mediated assessment: an exploratory LLM simulation and questionnaire study in university ideological and political education

**DOI:** 10.3389/fpsyg.2026.1908308

**Published:** 2026-10-30

**Authors:** Shaobo Liu, Weimin Liu

**Affiliations:** 1 School of Marxism, Central South University, Changsha, China; 2 School of Humanities, Anhui Polytechnic University, Wuhu, China

**Keywords:** argumentation, assessment validity, Generative AI, higher education assessment, task design

## Abstract

**Introduction:**

Generative AI weakens the correspondence between the final text of an assignment and the argumentative work involved in producing it. This study examined how task structure and AI-use instructions shape evidence use, engagement with objections, and revision in university ideological and political education.

**Methods:**

An LLM simulation crossed three task formats—position essays, evidence-integration memoranda, and follow-up-and-revision defense tasks—with three AI-use scenarios—simulated no-AI, responsible collaboration, and concealed ghostwriting. Two raters coded 148 generated texts with the Textual Response Artifact Scoring Index (TRASI), and an anonymous questionnaire collected 268 evaluations of 27 selected texts.

**Results:**

Responsible collaboration produced the highest overall rubric score (*M* = 3.32). Evidence-integration memoranda (*M* = 3.16) and follow-up-and-revision defenses (*M* = 3.10) exceeded position essays (*M* = 2.63). Position essays varied most across the three AI-use scenarios, whereas the memorandum and defense showed narrower differences because their instructions already required source explanation or response and revision. Questionnaire participants distinguished the same task functions: memoranda were rated highest for source–claim linkage, and defenses for counterargument response and revision explanation. Open-ended responses associated evidence integration, comparison of reasons, and revision with thinking, while formulaic structure, over-polished expression, and generic abstraction were associated with AI-like writing.

**Discussion:**

Task structure and AI-use instructions were functionally complementary. Structured tasks elicited source explanation or challenge and revision directly, so responsible collaboration added its largest contribution in the general position essay. GenAI-mediated assessment can therefore be designed around the argumentative decisions that writers are required to document.

## 1 Introduction

Generative AI has altered the evidential basis of university writing assessment. A polished response can combine a writer's decisions with model-assisted search, idea generation, drafting, rewriting, and language editing. The final prose may remain coherent even when the choices that shaped the argument are compressed or omitted. Valid assessment consequently depends on whether the task elicits the work relevant to the intended learning outcome, including how evidence was selected, how competing reasons were handled, and how a judgment changed (Dawson, 2020; Bearman et al., 2024; Dawson et al., 2024).

These demands are central to university ideological and political education. Written assignments in this field ask students to connect normative positions with factual material, compare accounts of public issues, respond to disagreement, and qualify judgments in light of social and institutional conditions. Three forms of argumentative work are especially important: constructing a case by connecting sources to claims; testing that case against objections or alternative explanations; and revising a judgment when the balance of reasons changes. A conventional position essay can present a persuasive conclusion while leaving much of this work implicit (Gruenhagen et al., 2024; Kofinas et al., 2025; Corbin et al., 2026).

Task design can require these activities to be articulated. An evidence-integration memorandum asks why particular sources were chosen and what each contributes to a claim. A follow-up-and-revision defense requires a response to challenge and an explanation of what was retained, qualified, or changed. Requirements for AI disclosure, verification, and the treatment of model suggestions add a second layer to the task by specifying how AI-assisted choices must be explained. Task architecture and AI-use governance consequently contribute different parts of the information included in the response.

The study investigates this complementarity in two stages. First, an LLM simulation crosses three task formats with three AI-involvement scenarios to compare the argumentative composition of the generated texts. Second, an anonymous questionnaire examines whether evaluators distinguish the intended functions of the tasks and which textual cues guide their judgments. TRASI serves as an author-developed coding rubric for five textual features: process visibility, source–claim linkage, contextual grounding, counterargument handling, and revision-rationale clarity. The analysis asks how task format and AI-use instructions shape these features, how the two design components interact, and how evaluators respond to the resulting differences.

## 2 Related research

### 2.1 Assessment validity and the evidence provided by written work

Assessment validity depends on the inference supported by the performance a task elicits. Constructive alignment connects learning outcomes, learning activities, and assessment requirements, while formative assessment uses performance information to identify the distance between current work and the expected standard (Biggs, 1996; Sadler, 1989; Black and Wiliam, 1998). A writing task therefore shapes validity through its demands: a request for a clear conclusion elicits one kind of performance, while requirements to compare sources, justify their use, answer objections, and revise a position elicit others.

Scoring criteria reinforce this elicitation function. Rubrics and feedback focus attention on particular qualities of performance and contribute to evaluative judgment (Nicol and Macfarlane-Dick, 2006; Boud and Soler, 2016; Jonsson and Svingby, 2007; Panadero and Jonsson, 2013). When evidence evaluation or revision is an intended outcome, the assignment must ask writers to explain source choice, evidential relevance, competing reasons, and changes in their conclusion. GenAI makes this alignment more consequential because fluency and formal completeness can be produced while those decisions remain unstated (Kaldaras et al., 2024).

Dawson's account of valid inference places the warrant between performance and interpretation at the center of assessment design (Dawson, 2020). The central question is what features of the response justify the conclusion an evaluator wishes to draw. In GenAI-mediated writing, this shifts attention toward task elicitation: the assignment must generate sufficiently specific information about evidence construction, argumentative testing, and revision for those aspects of performance to be evaluated.

### 2.2 GenAI, academic integrity, and task redesign

Academic integrity research has long examined how plagiarism, contract cheating, and third-party authorship disrupt the connection between submitted work and the performance attributed to a student (McCabe et al., 2001; Newton and Lang, 2016). LLMs intensify this problem by producing original prose that may evade conventional similarity matching and by participating across multiple stages of writing (Weber-Wulff et al., 2023). The design problem now includes both governance of tool use and the structure of the assignment itself.

University policies increasingly specify permitted uses, disclosure, and responsibility, while assessment redesign determines what writers must explain about source selection, verification, and revision (Chan, 2023; Moorhouse et al., 2023; Bower et al., 2024; Luo, 2024; Gonsalves, 2025). These functions are connected. Disclosure identifies where GenAI contributed; task requirements determine whether the response explains how suggestions were checked, compared with evidence, accepted, modified, or rejected.

Recent reviews accordingly emphasize assessment arrangements that retain clear responsibility for decisions made within AI-supported work (Lo, 2023; Tlili et al., 2023; Farrokhnia et al., 2024; Weng et al., 2024; Xia et al., 2024). For text-based assignments, responsibility is expressed through the quality of source use, the handling of conflicting information, and the rationale for revision. AI-use rules become educationally consequential when they are integrated into tasks that require those decisions to be explained.

### 2.3 Argumentation and value judgment in ideological and political education

Ideological and political education frequently uses contested public questions to develop reasoned judgment. Work on civic education and public reason emphasizes articulating premises, exchanging reasons, and justifying decisions to others (Gutmann, 1999; Biesta, 2009; Biskamp, 2026). Strong performance therefore depends on more than stating a position: writers must connect claims with relevant grounds, consider the conditions under which a judgment holds, and engage seriously with alternatives.

Argument construction begins with the organization of evidence. Toulmin's distinction among claims, grounds, warrants, and qualifiers shows why citing a source is insufficient unless its contribution to the conclusion is explained (Toulmin, 2003). Studies of argumentation similarly emphasize the selection, coordination, and justification of evidence (Berland and Reiser, 2009; Osborne, 2010). An evidence-integration task strengthens this demand by assigning each source a role in the argument.

Arguments are also tested through challenge and revised as reasons change. Counterarguments expose weaknesses, competing explanations, and limits in an initial position; responding to them requires comparison and qualification, not simple rebuttal (Kuhn, 1991; Nussbaum and Schraw, 2007; Osborne, 2010). Revision extends this process across time by requiring the writer to explain what changed and why (Flower and Hayes, 1981; Nicol and Macfarlane-Dick, 2006; Chinn and Brewer, 1993). Evidence construction, challenge, and revision are therefore related but distinct forms of argumentative work that task formats can emphasize in different combinations.

### 2.4 LLM simulation as task pretesting

LLMs can generate comparable sets of responses from common source materials while task instructions and AI-use scenarios are varied systematically (Bommasani et al., 2021; Wei et al., 2022). This creates a controlled setting for examining whether a prompt elicits its intended features and whether alternative task designs produce different response profiles. Known tendencies of LLM output, including hallucination, stylistic regularity, and plausible but weakly grounded explanation, also make source use and argumentative specificity important objects of analysis (Bender et al., 2021; Ji et al., 2023).

Used in this way, simulation functions as task pretesting. It allows researchers to compare a general essay with tasks that explicitly require source explanation, challenge, or revision, and to examine whether AI-use instructions add the same information across those formats. Evaluator judgments provide a second perspective on whether the designed differences are recognizable. Combining controlled generation with human evaluation brings task elicitation, AI-use governance, and argumentative quality into a single design.

## 3 Analytical framework and research expectations

### 3.1 Argumentative processes and their operationalization

The analytical framework distinguishes three forms of argumentative work. Evidence construction concerns how sources are selected, interpreted, and connected to claims. Challenge concerns how a position engages with objections, competing explanations, or conflicting evidence. Revision concerns how a judgment is retained, qualified, or changed after challenge. Contextual analysis runs through all three because the force of evidence, objections, and revisions depends on the actors, institutions, constraints, and conditions of the issue (Toulmin, 2003; Berland and Reiser, 2009; Osborne, 2010; Brown et al., 1989; Gulikers et al., 2004; Kuhn, 1991; Nussbaum and Schraw, 2007; Flower and Hayes, 1981; Nicol and Macfarlane-Dick, 2006; Chinn and Brewer, 1993).

TRASI translates these processes into five coding categories. Evidence anchoring (EA) codes the connection between sources and claims; counterargument handling (CA) codes engagement with an opposing position; and revision-rationale clarity (RR) codes what was retained or changed and why. Process visibility (PV) summarizes how clearly the response connects information selection, comparison, and judgment, while contextual embeddedness (SC) codes attention to the concrete conditions of the issue. The five scores are averaged for an overall profile and retained separately to show which aspect of argumentative work each task emphasizes.

### 3.2 Expected effects of task structure and AI-use instructions

The three task formats distribute argumentative demands differently. A position essay asks for a stance and supporting reasons. An evidence-integration memorandum adds source selection and explanation of each source's contribution. A follow-up-and-revision defense introduces objections after the initial response and asks the writer to explain the resulting defense and revision. The memorandum should therefore emphasize evidence construction, while the defense should emphasize challenge and judgment updating.

The AI-involvement scenarios add a second set of requirements. The simulated no-AI prompt portrays completion without GenAI. Responsible collaboration requires disclosure, verification, comparison with the writer's judgment, and explanation of revisions. Concealed ghostwriting requests a complete response without disclosure of tool involvement. Responsible collaboration should therefore elicit more information about verification and revision than the other scenarios.

Task structure is expected to moderate this scenario effect. A general position essay leaves source handling, challenge, and revision largely unspecified, so responsible-collaboration instructions should produce a comparatively large change. The memorandum and defense already require part of that work and should depend less on the AI-use prompt. This functional complementarity provides the basis for the task-by-scenario comparison.

## 4 Methods

### 4.1 Research design

The study combined an LLM simulation with an anonymous questionnaire (Figure 1). The simulation crossed three task formats, three AI-involvement scenarios, and three thematic domains: public responsibility and rule consciousness; social participation and value judgment; and national narratives and analysis of real-world issues. Generated texts were screened and rated with TRASI. The questionnaire presented a balanced subset to respondents, who evaluated the argumentative features, perceived assessment diagnosticity, AI-use transparency, and suitability for formal assessment. This design enabled systematic comparison of task requirements, AI-use instructions, and evaluator judgments.

**Figure 1:**
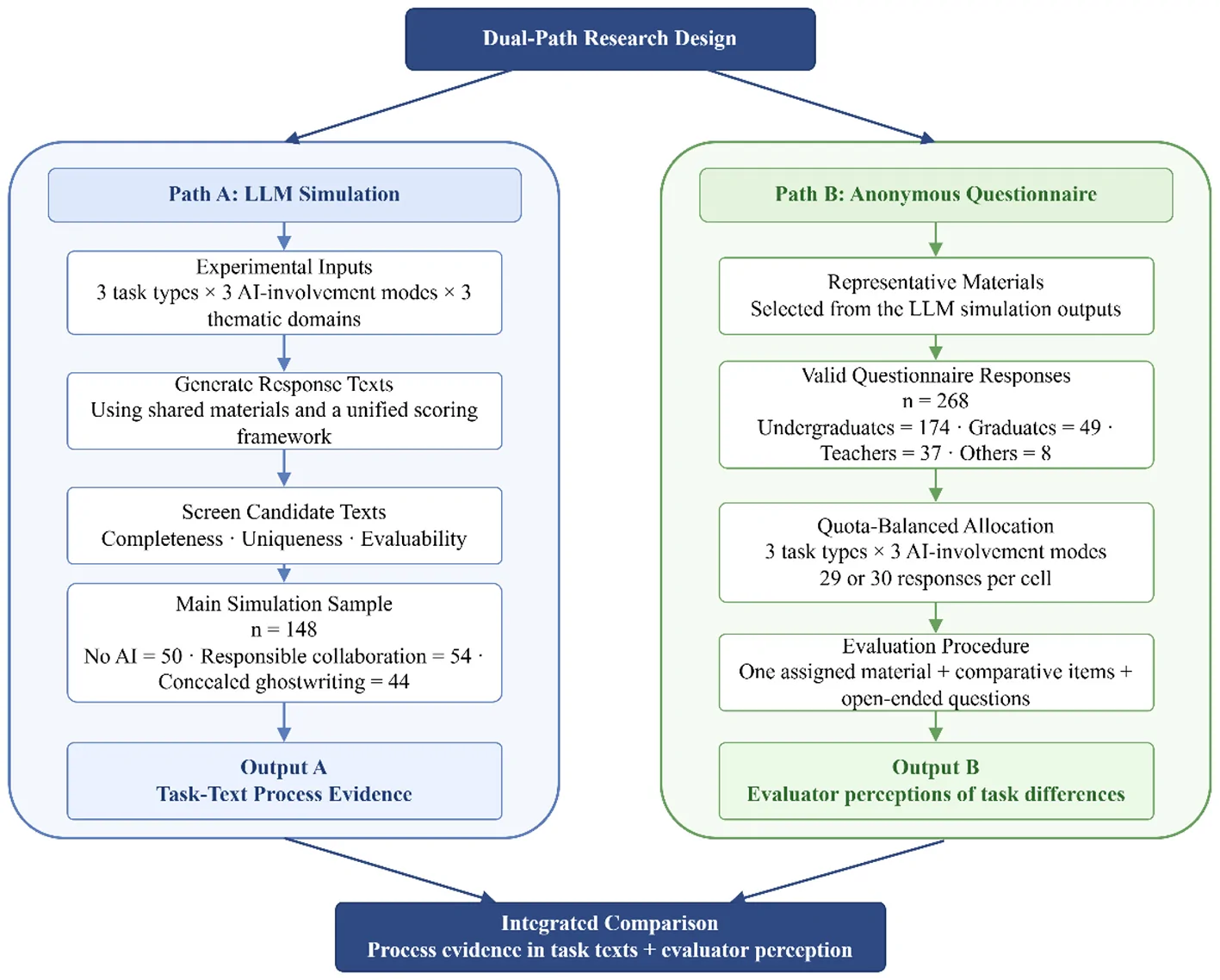
Flowchart of the simulation, artifact scoring, stimulus selection, and questionnaire stages.

Of 189 generated candidates, 41 were excluded before formal scoring: 14 were incomplete, 13 were duplicate or highly similar outputs, and 14 did not contain enough material for reliable evaluation. The final simulation sample contained 148 texts: 50 in simulated no-AI, 54 in responsible collaboration, and 44 in concealed ghostwriting; and 43 position essays, 59 evidence-integration memoranda, and 46 follow-up-and-revision defenses. The condition-by-domain screening results are reported in Supplementary Tables S4–S7.

### 4.2 Generation and stimulus selection

The texts were generated with DeepSeek-V3.1 through the official DeepSeek API using the deepseek-chat endpoint between 18 and 24 March 2026. Generation used temperature 0.70, top-p 0.90, a maximum of 2,200 output tokens, and zero frequency and presence penalties. Web browsing, external retrieval, retrieval-augmented generation, and tool calling were disabled. Separate sessions were used for candidates, and responses were repeated only after a technical failure. Full prompts, source packages, generation settings, candidate identifiers, and screening results are provided in the Supplementary material.

Questionnaire stimuli were selected before formal TRASI scoring. One retained text was selected from each thematic domain within every task-by-scenario cell, producing 27 stimuli. Eligible texts had to be complete, nonduplicative, within the planned length range, consistent with the assigned scenario, based only on the supplied materials, free of obvious factual hallucination, and sufficiently developed for evaluation. Each cell therefore contained three different texts. Respondents saw the task instructions, the assigned text, and necessary background information, but not the formal condition labels.

### 4.3 Task conditions and text scoring

The position essay asked for a judgment on a public issue and reasons supporting that judgment. The evidence-integration memorandum required the response to use multiple supplied sources, connect each source to a claim, and justify source selection. The follow-up-and-revision defense added standardized questions after the initial response and required a revised defense that addressed objections and explained any change in judgment. Across all task types, the simulated no-AI prompt portrayed completion without GenAI; responsible collaboration required disclosure, verification, and explanation of model-assisted revisions; concealed ghostwriting required a complete response without acknowledging AI involvement.

Each text received a 1–5 anchored score on PV, EA, SC, CA, and RR, and the mean of the five scores formed the overall TRASI value. Two human raters independently scored all 148 texts. One rater had doctoral training in ideological and political education and experience evaluating argumentative assignments; the other had doctoral training in educational psychology or evaluation and experience with rubric-based text analysis. Both completed approximately two hours of training and two calibration rounds using 20 pilot texts that were excluded from the analytical sample. Formal texts were relabeled T001–T148, condition labels were withheld, and rating order was randomized separately for each rater.

Inter-rater reliability was calculated from the unreconciled ratings. Quadratic-weighted Cohen's κ was estimated for each dimension with 2,000 artifact-level bootstrap resamples, and overall-score agreement was estimated with two-way random-effects, absolute-agreement ICC(2,1) and ICC(2,2). One-point differences were discussed with reference to the rubric; larger or unresolved differences were adjudicated by a third educational researcher. The reconciled scores were used for substantive analyses. The dimensional definitions appear in Supplementary Table S8, the complete anchored rubric in Supplementary Table S9, and reliability and adjudication procedures in Supplementary Table S10.

### 4.4 Questionnaire participants and ethics

The questionnaire was administered through Wenjuanxing from 2 to 20 April 2026. Participants were recruited voluntarily through course, year-level, graduate-student, and faculty groups at Central South University. No commercial panel or paid sample service was used. The final sample comprised 174 undergraduates (64.9%), 49 graduate students (18.3%), 37 university teachers (13.8%), and 8 other university-affiliated respondents (3.0%). The nine task-by-scenario cells contained 29 or 30 respondents each, and every respondent evaluated one artifact.

Participants had to be at least 18 years old, affiliated with the university, able to read the Chinese materials, provide electronic informed consent, read the assigned artifact, and complete the core measures. Of 289 records, 8 incomplete submissions, 7 records that failed at least one instructed-response attention check, and 6 records completed in under 6 minutes were excluded before analysis. No material incentives were offered. Recruitment, screening, respondent background, course experience, and GenAI-use distributions are reported in Supplementary Tables S11–S14.

The study used an anonymous, minimum-risk educational survey procedure under Central South University provisions. The consent page described the purpose, procedure, voluntary nature of participation, right to withdraw before submission, anonymous analysis, and aggregate reporting. No directly identifying information was requested or retained.

### 4.5 Questionnaire measures and open-ended coding

All quantitative items used a five-point scale from 1 (strongly disagree) to 5 (strongly agree). Fifteen items assessed the five perceived TRASI categories, with three items per category; the overall perceived score was the mean of all 15 items. Six items assessed perceived assessment diagnosticity, five assessed AI-use transparency and ethical acceptability, and six assessed learning value and support for reform. Six additional items served as task and scenario checks: multiple-source integration (B1), explanation of evidence selection (B2), response and revision after questioning (B3), AI-use disclosure (B4), verification of AI-provided information (B5), and explanation of accepted, modified, or rejected AI suggestions (B6). Full item wording is provided in Supplementary Table S17.

Comparative items asked which task best displayed a thinking process and which was most suitable for formal assessment. Four open-ended questions asked respondents to identify evidence of thinking (H1), formulaic or AI-like cues (H2), useful additions to the task (H3), and priorities for assessment reform (H4). Responses were coded with a predefined multi-label dictionary. The coding unit was the smallest meaningful proposition, and a response could receive several codes. Two trained coders independently coded all usable responses after a two-hour training session and calibration on 30 pilot responses. Theme-level Cohen's κ was calculated before reconciliation; unresolved disagreements were adjudicated by a third researcher. The question structure, coding dictionaries, theme frequencies, and reliability estimates appear in Supplementary Tables S19–S23. The retained qualitative dataset contains coded responses; original verbatim text is not present in the retained study archive for quotation verification.

### 4.6 Statistical analysis

Simulation results were summarized with means, confidence intervals, and dimensional profiles. A two-way analysis of variance tested simulation scenario, task format, and their interaction. Table 1 reports ordinary η^2^, calculated relative to total variation, with 95% confidence intervals from 5,000 bias-corrected and accelerated bootstrap resamples at the artifact level. Prespecified pairwise contrasts used Welch comparisons and Cohen's *d*; the Figure 2 intervals for these contrasts used 5,000 bias-corrected and accelerated (BCa) bootstrap resamples, with the simulated artifact as the resampling unit and seed 20260324. Pearson correlations among rubric dimensions used Fisher *z* confidence intervals.

**Table 1:** Two-way analysis of variance for TRASI scores in the 148 simulated artifacts.

Factor	df	*F*	*p*	η^2^	95% CI
Simulation scenario	2	21.52	< 0.001	0.192	[0.095, 0.286]
Task type	2	14.77	< 0.001	0.131	[0.051, 0.224]
Simulation scenario × Task type	4	3.25	0.014	0.058	[0.010, 0.128]

**Figure 2:**
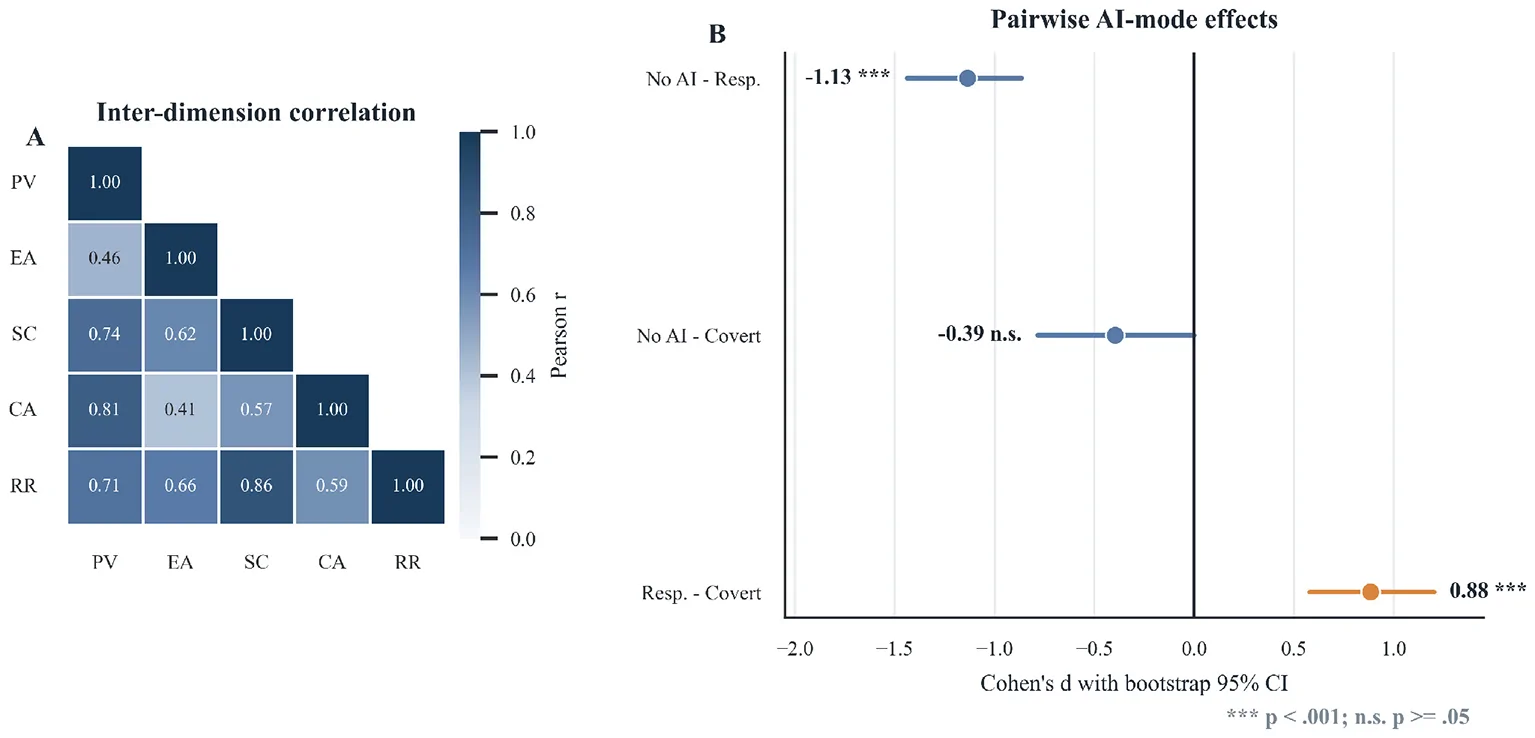
Relations among the five TRASI categories and pairwise scenario contrasts. Panel **(A)** presents Pearson correlations. Panel **(B)** presents Cohen's *d* with BCa bootstrap 95% confidence intervals; negative values indicate a higher mean for the second scenario in the contrast.

For the questionnaire, internal consistency was assessed for the perceived-feature items. The 15-item total had α = 0.816, and the three-item category scores had α = 0.396–0.559; these category scores were used for descriptive profiles and exploratory task-function comparisons. Two-way analyses of variance tested task format, scenario, and their interaction for the main perception outcomes, with partial η^2^ and 95% confidence intervals. Prespecified contrasts used Cohen's *d*. A sensitivity analysis repeated the principal comparisons after excluding respondents who answered at least two condition-relevant check items in the direction opposite to the assigned manipulation. Pearson correlations and multiple linear regression examined associations between perceived features and perceived assessment diagnosticity. Because the category scores had limited internal consistency, the regression coefficients were treated as exploratory conditional associations whose magnitudes may reflect measurement error. Open-ended results are reported as multi-label frequencies using the number of usable responses to each question as the denominator.

## 5 Results

### 5.1 Overall differences across task and AI-involvement conditions

AI-use scenario produced the largest overall difference in the generated texts. Responsible collaboration had the highest TRASI mean (*M* = 3.32, *n* = 54), followed by concealed ghostwriting (*M* = 2.93, *n* = 44) and simulated no-AI (*M* = 2.67, *n* = 50; Figure 3A). The responsible-collaboration contrast was large relative to simulated no-AI (*d* = −1.13, 95% CI [−1.54, −0.72]) and substantial relative to concealed ghostwriting (*d* = 0.88, 95% CI [0.46, 1.30]). The simulated no-AI and concealed-ghostwriting estimates were closer (*d* = −0.39, 95% CI [−0.80, 0.02]). The largest separation was associated with the disclosure, verification, and revision requirements in the responsible-collaboration prompt.

**Figure 3:**
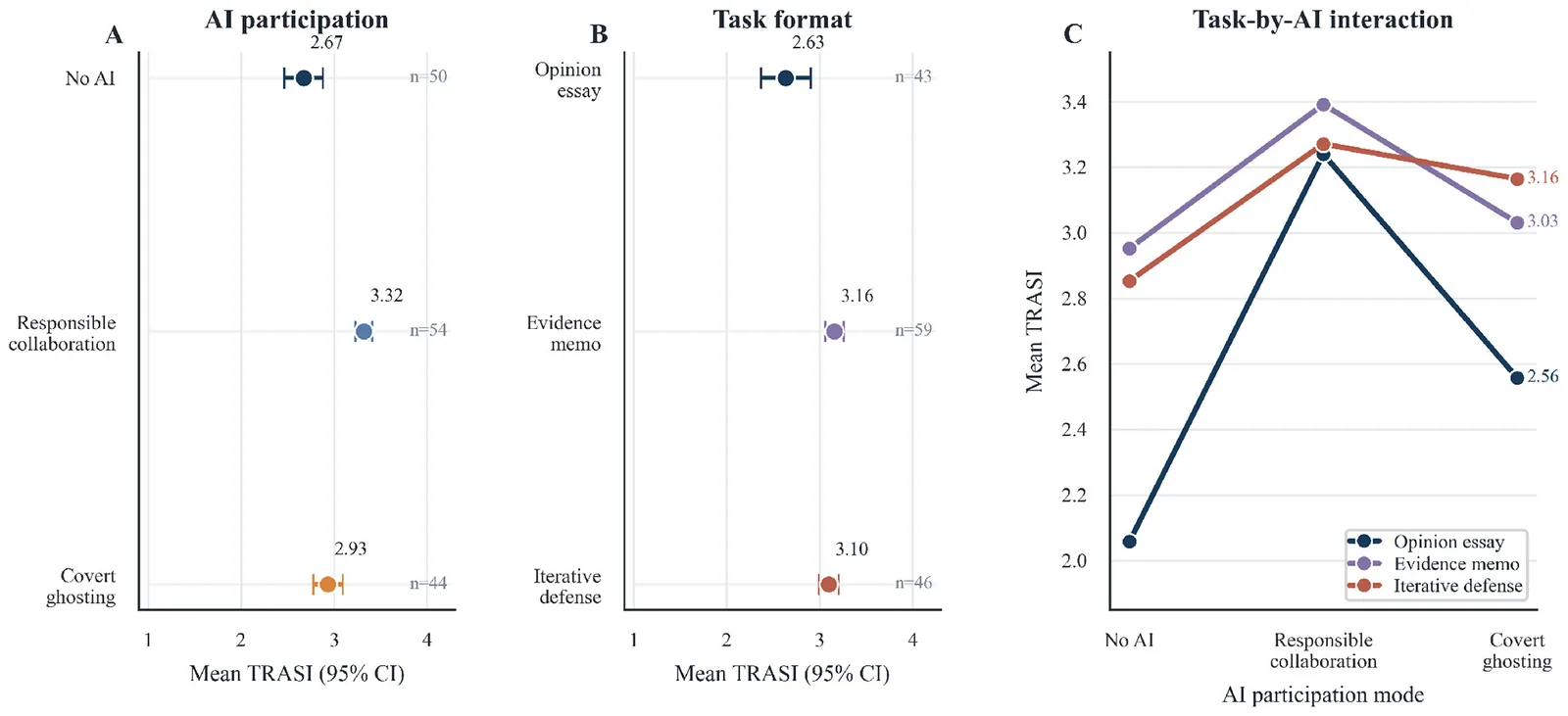
Overall TRASI scores across AI-involvement scenarios and task formats. Panel **(A)** shows scenario means and 95% confidence intervals; Panel **(B)** shows task-format means and 95% confidence intervals; Panel **(C)** shows the task-by-scenario pattern.

Task format produced a second ordering. Evidence-integration memoranda had the highest mean (*M* = 3.16, *n* = 59), followed by follow-up-and-revision defenses (*M* = 3.10, *n* = 46), while position essays were lower (*M* = 2.63, *n* = 43; Figure 3B). The two structured formats produced higher scores across the AI-use scenarios, with the memorandum emphasizing source use and the defense emphasizing response and revision.

The interaction concentrated on the position essay. Its mean rose from 2.06 in simulated no-AI to 3.23 in responsible collaboration and fell to 2.56 in concealed ghostwriting. Evidence-integration memoranda showed a narrower spread (2.95, 3.39, and 3.03), while follow-up-and-revision defenses remained comparatively high under responsible collaboration (3.27) and concealed ghostwriting (3.16). The structured tasks already called for source explanation or response and revision, so the responsible-collaboration prompt supplied its largest additional contribution in the general position essay (Figure 3C).

Simulation scenario accounted for more total variation than task format (ordinary η^2^ = 0.192, 95% CI [0.095, 0.286] vs. η^2^ = 0.131, 95% CI [0.051, 0.224]), while their interaction was smaller and still meaningful (η^2^ = 0.058, 95% CI [0.010, 0.128]; Table 1). The effect sizes place the main difference at the scenario level, with task format adding a separate contribution and the interaction arising primarily from the position essay's sensitivity to AI-use instructions.

### 5.2 Dimensional sources of the overall pattern

The five dimensions did not respond uniformly to the task and scenario conditions. Contextual grounding was high in every scenario (3.48 in simulated no-AI, 4.11 in responsible collaboration, and 3.82 in concealed ghostwriting), consistent with prompts organized around concrete public issues. Source–claim linkage and counterargument response were lower in simulated no-AI (both *M* = 2.26) and increased when the task or scenario requested them. Revision explanations followed the same dependence on explicit requirements. The largest differences therefore concerned evidence use, challenge, and revision, whereas contextual grounding changed less across conditions (Figure 4).

**Figure 4:**
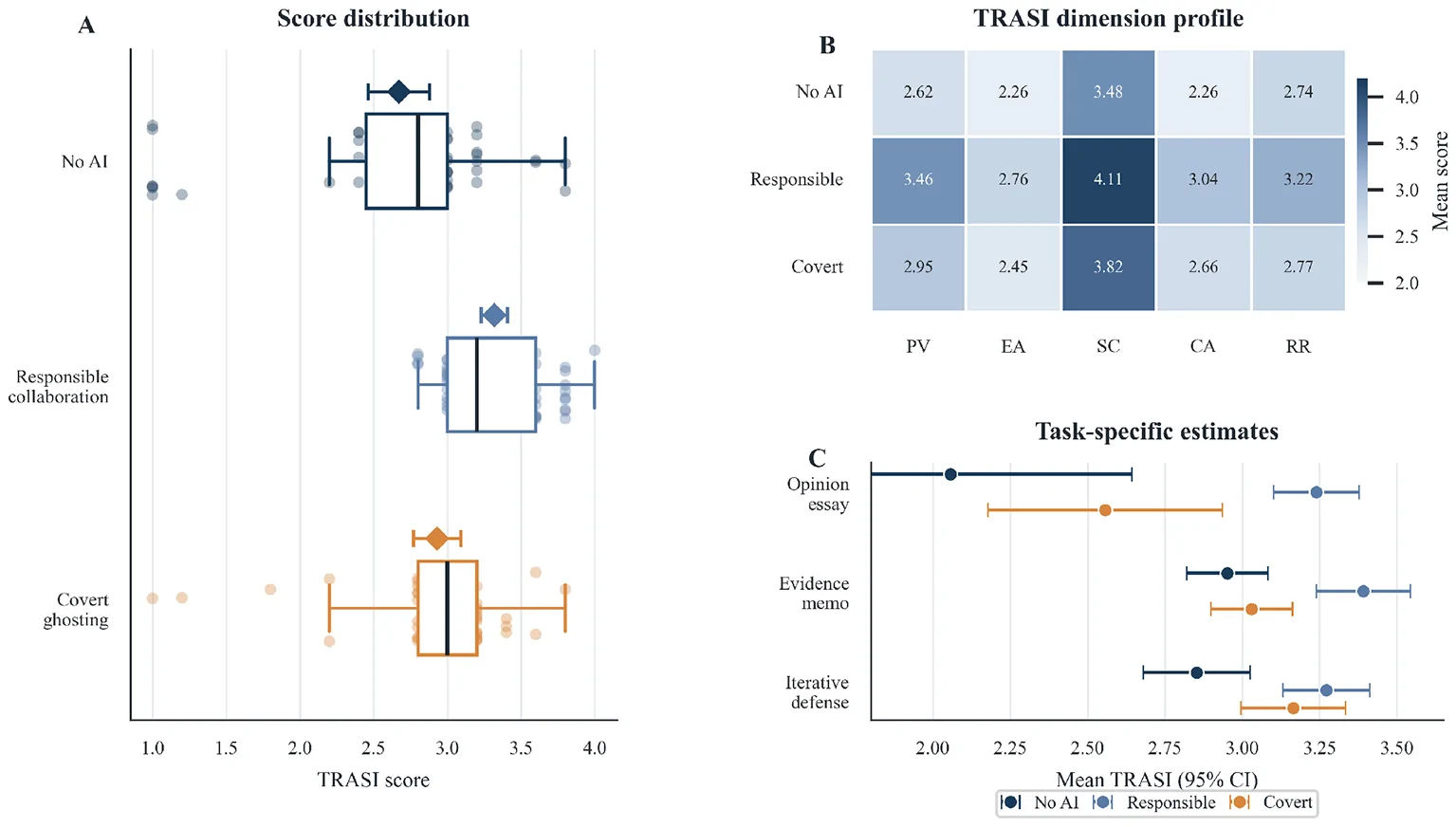
Distribution and dimensional profile of TRASI scores. Panel **(A)** presents artifact-level distributions by scenario; Panel **(B)** presents mean scores for the five operational categories; Panel **(C)** presents task-specific scenario estimates with 95% confidence intervals.

Task-specific estimates followed the same division. Evidence-integration memoranda maintained similar overall means across simulated no-AI and concealed ghostwriting, and follow-up-and-revision defenses remained high across scenarios. Position essays showed the widest movement because their general stance-and-reasons format left more of the argumentative work unspecified.

The five dimensions also formed distinct clusters within the generated texts (Figure 2A). Contextual grounding and revision explanation were strongly associated (*r* = 0.86), as were process visibility and counterargument response (*r* = 0.81). Source–claim linkage and counterargument response had the weakest association (*r* = 0.41), indicating that evidence use and objection handling were related but separate demands. The pairwise contrasts in Figure 2B again placed the largest separation around responsible collaboration, with a closer simulated no-AI vs. concealed-ghostwriting comparison.

### 5.3 Whether evaluators distinguished the task structures

Participant ratings reproduced the main task and scenario ordering. Follow-up-and-revision defenses received the highest overall perceived-feature score (*M* = 3.12, *n* = 90), followed by evidence-integration memoranda (*M* = 3.07, *n* = 88) and position essays (*M* = 2.72, *n* = 90). Responsible collaboration again ranked highest across scenarios (*M* = 3.29, *n* = 90), followed by concealed ghostwriting (*M* = 2.91, *n* = 89) and simulated no-AI (*M* = 2.70, *n* = 89; Figures 5A, B). The main inferential statistics for the questionnaire outcomes are summarized in Table 2.

**Figure 5:**
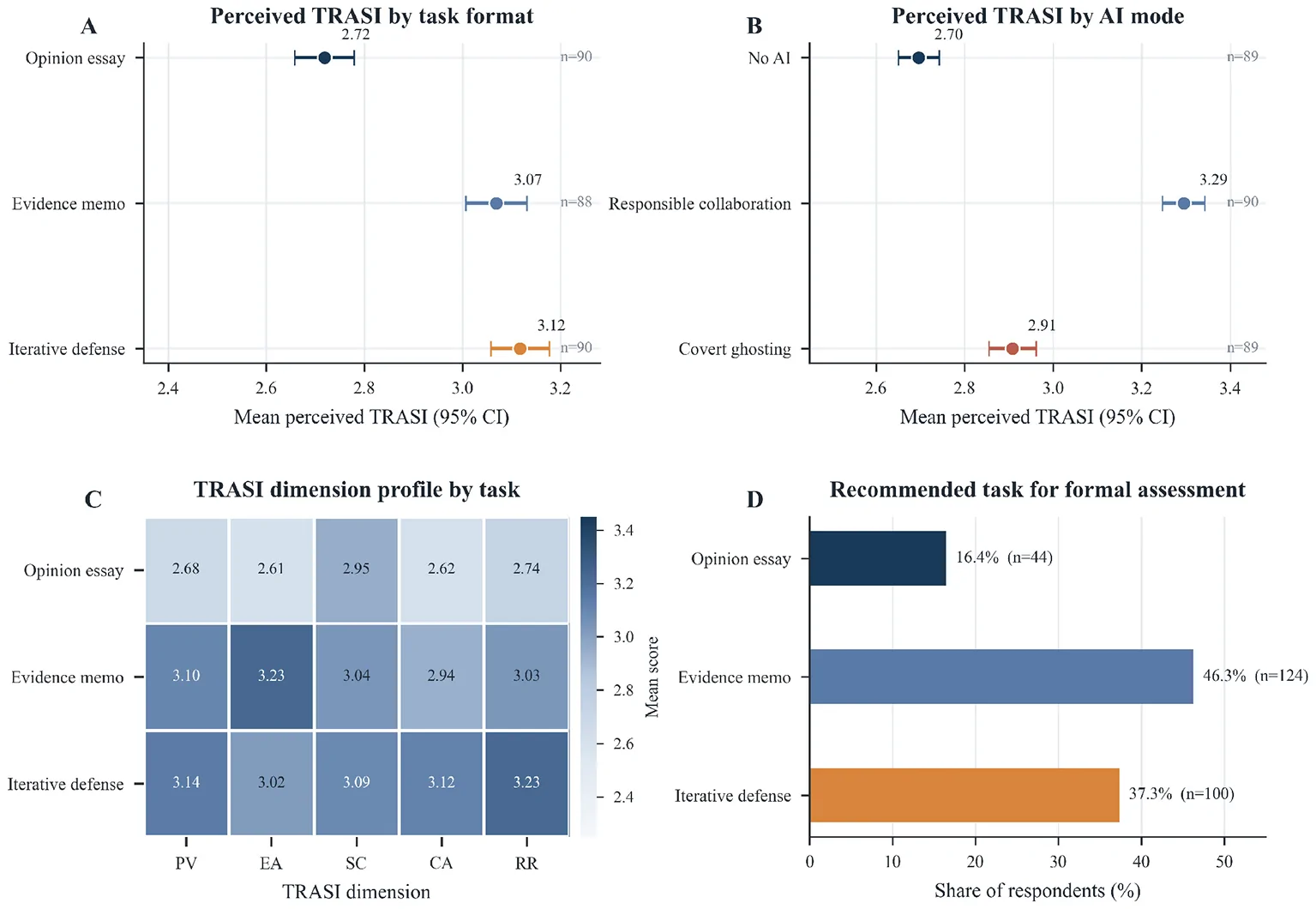
Evaluator perceptions of the selected simulated artifacts. Panels **(A)** and **(B)** show overall perceived-feature means by task and scenario. Panel **(C)** shows task-specific dimensional profiles. Panel **(D)** shows the task selected as most suitable for formal assessment.

**Table 2:** Main inferential statistics from the anonymous questionnaire.

Outcome variable	Factor	df	*F*	Partial η^2^	95% CI
Perceived TRASI	Task type	2, 259	172.72***	0.572	[0.498, 0.632]
Perceived TRASI	Simulation scenario	2, 259	336.53***	0.722	[0.670, 0.763]
Perceived TRASI	Task × Scenario	4, 259	1.29	0.019	[0.000, 0.051]
Perceived assessment diagnosticity	Task type	2, 259	47.02***	0.266	[0.178, 0.349]
Perceived assessment diagnosticity	Simulation scenario	2, 259	81.96***	0.388	[0.299, 0.465]
Support for reform	Task type	2, 259	94.80***	0.423	[0.335, 0.498]
Support for reform	Simulation scenario	2, 259	41.76***	0.244	[0.157, 0.326]
AI transparency and ethical acceptability	Simulation scenario	2, 259	4.81**	0.036	[0.002, 0.086]

*p < 0.05, ** *p* < 0.01, ***p < 0.001.

The contrasts were large for both task and scenario comparisons. Relative to position essays, ratings were higher for evidence-integration memoranda (*d* = 1.18, 95% CI [0.86, 1.50]) and follow-up-and-revision defenses (*d* = 1.37, 95% CI [1.05, 1.69]). Responsible collaboration exceeded simulated no-AI by *d* = 2.63 (95% CI [2.23, 3.03]) and concealed ghostwriting by *d* = 1.58 (95% CI [1.24, 1.92]); concealed ghostwriting also exceeded simulated no-AI (*d* = 0.89, 95% CI [0.58, 1.20]). These contrasts followed the additional disclosure, verification, and revision information required in the responsible-collaboration materials.

Dimension-specific judgments followed the function of each format. Evidence-integration memoranda were rated highest on source–claim linkage (*M* = 3.23), while follow-up-and-revision defenses were rated highest on counterargument response (*M* = 3.12) and revision explanation (*M* = 3.23). Position essays were lower on each category. Formal-assessment choices followed the same distinction: 46.3% selected the memorandum, 37.3% selected the defense, and 16.4% selected the position essay (Figures 5C, D).

Manipulation checks aligned with the intended task functions. The memorandum was highest on multiple-source integration and explanation of evidence selection (partial η^2^ = 0.513 and 0.525), the defense was highest on response to questioning and revision (partial η^2^ = 0.705), and responsible collaboration was distinguished on AI disclosure, verification, and explanations of accepted or rejected suggestions (partial η^2^ = 0.868, 0.777, and 0.776). Full means and item-level tests are reported in Supplementary Tables S17–S18. Nine respondents met the prespecified nonpassing criterion; after their exclusion, the ordering of task and scenario means was preserved and effect estimates remained in the same substantive range.

The five perceived categories jointly explained 58.0% of the variance in assessment diagnosticity (*R*^2^ = 0.580, adjusted *R*^2^ = 0.572). Counterargument response had the largest coefficient (*B* = 0.24, 95% CI [0.14, 0.34]), followed by process visibility (*B* = 0.21, 95% CI [.11, .31]), source–claim linkage (*B* = 0.18, 95% CI [0.10, 0.26]), and revision explanation (*B* = 0.17, 95% CI [0.07, 0.27]). Contextual grounding had a small coefficient whose interval included zero (*B* = 0.09, 95% CI [−0.01, 0.19]). Given the limited internal consistency of the category scores, these coefficients are exploratory conditional associations and their relative magnitudes may be affected by measurement error. Collinearity and residual diagnostics are reported in Supplementary Table S25.

### 5.4 Textual cues underlying evaluator judgments

The open-ended responses separated two bases for judgment. When respondents described evidence of thinking, they focused on evidence integration (158 of 262 usable responses, 60.3%), comparative reasoning (131, 50.0%), revision rationale (116, 44.3%), contextual grounding (101, 38.5%), and responses to counterarguments (94, 35.9%). When they described AI-like writing, they focused on formulaic structure (146 of 258, 56.6%), over-polished expression (118, 45.7%), unsupported conclusions (103, 39.9%), generic abstraction (91, 35.3%), repetitive patterns (82, 31.8%), and vague attribution (74, 28.7%).

Suggestions for assessment reform specified the additions respondents wanted in future tasks. Among 261 usable H3 responses, the most frequent proposals were follow-up questioning (149, 57.1%), draft or revision records (137, 52.5%), evidence tables (121, 46.4%), reasons for source selection (112, 42.9%), and AI-use disclosure (96, 36.8%). Among 256 usable H4 responses, the leading priorities were process-oriented task design (151, 59.0%) and scoring-criteria reform (133, 52.0%), followed by AI-use rules (111, 43.4%), follow-up or defense (98, 38.3%), and multi-stage submission (87, 34.0%). The two response patterns connect the quantitative findings with distinct evaluative cues: argumentative organization was used to identify thinking, whereas regularity and weak specificity were used to identify AI-like writing (Figure 6).

**Figure 6:**
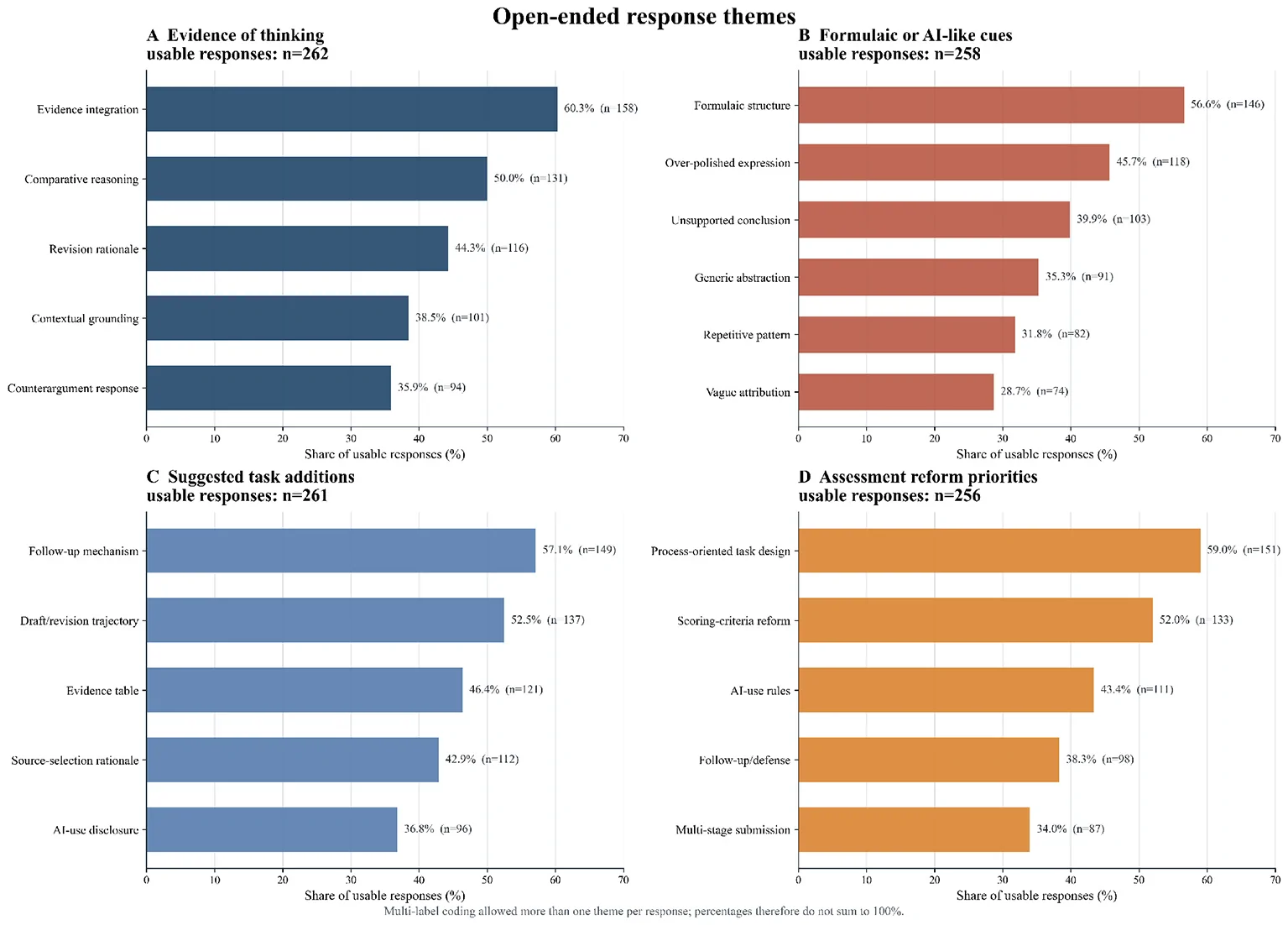
Themes in the four open-ended questions. Bar length represents the percentage of usable responses assigned each code, and labels report both the percentage and theme count. Responses could receive more than one code. **(A)** Evidence of thinking usable responses: *n* = 262. **(B)** Formulaic or AI-like cues usable responses: *n* = 258. **(C)** Suggested task additions usable responses: *n* = 261. **(D)** Assessment reform priorities usable reform usable responses: *n* = 256.

## 6 Discussion

### 6.1 Task structure as an externalization constraint

Task architecture constrained the argumentative work a response had to carry. A position essay asks for a conclusion and supporting reasons, so source choice, responses to objections, and revision can remain compressed in the prose. The memorandum assigns sources a role in the case, and the defense introduces challenge followed by a revised position. These requirements give evidence use, challenge, and judgment updating a defined place in the submitted performance.

This mechanism extends constructive alignment to GenAI-mediated writing. Alignment links intended outcomes with activities and with the evidence produced by assessment (Biggs, 1996; Dawson, 2020). When evidence evaluation or judgment revision matters, a source-selection rationale, response to an objection, or explanation of a changed conclusion makes that activity part of the performance sample. The assignment therefore contributes directly to the validity argument by shaping the evidence from which an evaluator forms a judgment.

### 6.2 Task structure and AI-use instructions are functionally interdependent

AI-use instructions contributed different amounts of information across task formats. Responsible collaboration produced the largest change for the position essay, where disclosure, verification, and revision requirements supplied operations absent from the general prompt. The memorandum and defense changed less across scenarios because source explanation or response and revision were already built into their formats. Task architecture and AI-use governance consequently operate as complementary design components: the base task carries some argumentative work, and the AI-use condition adds what remains unspecified.

This complementarity clarifies how institutional AI policies connect with assessment practice. Disclosure rules identify responsibility for tool use, while task requirements determine what that responsibility entails in the response: checking a suggestion, comparing it with supplied evidence, or explaining why a judgment changed (Chan, 2023; Moorhouse et al., 2023; Bower et al., 2024; Luo, 2024; Gonsalves, 2025). Governance becomes consequential when it is attached to a task that gives these decisions a substantive role.

### 6.3 AI-use requirements as part of task structure

The evidence-integration memorandum and the follow-up-and-revision defense organize different kinds of argument. The memorandum assigns sources an evidential function, requiring the writer to connect grounds with claims and to explain why a source belongs in the case. This is the practical counterpart of Toulmin's distinction among grounds, warrants, and claims (Toulmin, 2003; Berland and Reiser, 2009; Osborne, 2010). The defense organizes argument as a response to pressure: a follow-up objection requires the writer to represent an alternative, evaluate the force of the challenge, and state what remains or changes in the original conclusion. The strong associations of process visibility with counterargument response (*r* = 0.81) and contextual grounding with revision explanation (*r* = 0.86), together with the weaker association between source–claim linkage and counterargument response (*r* = 0.41), show that evidence organization and challenge handling are related but distinct demands.

Responsible collaboration adds another layer to this task architecture. Disclosure identifies where GenAI entered the work; verification and revision requirements specify what the writer must say about checking a suggestion, comparing it with supplied evidence, and accepting, modifying, or rejecting it (Chan, 2023; Moorhouse et al., 2023; Bower et al., 2024; Luo, 2024; Gonsalves, 2025). The largest scenario difference for position essays reflects the absence of equivalent requirements in the base task. The structured formats already carried part of this burden, so the same AI-use instructions added less to their responses.

### 6.4 Evaluator judgments respond to argumentative structure

Participant judgments followed the functions built into the task formats. Memoranda received the highest ratings for source–claim linkage, whereas defenses received the highest ratings for counterargument response and revision explanation. In the exploratory regression, perceived assessment diagnosticity was most closely associated with how a response handled objections, organized the path to a judgment, connected sources with claims, and explained revision. Contextual grounding contributed less once those features were considered. The judgments therefore tracked the organization of the argument alongside its linguistic presentation.

The open-ended coding specifies the cues behind these judgments. Evidence integration, comparison of reasons, and revision rationale were the most common features associated with thinking. Descriptions of AI-like writing focused on formulaic structure, over-polished expression, generic abstraction, repetition, and vague attribution. One set of cues concerns how a position is built and tested; the other concerns regularity and specificity in the prose. The reform suggestions point to the same design implications, emphasizing follow-up questions, draft histories, evidence tables, source-selection rationales, and AI-use disclosure as ways of structuring the material submitted for evaluation (Sadler, 1989; Nicol and Macfarlane-Dick, 2006).

## 7 Limitations and future research

The controlled generation conditions isolated variation in task requirements and AI-use instructions, while simplifying the complexity of classroom writing. Students bring prior knowledge, course experience, and different interpretations of a task, and their arguments develop through iterative choices about evidence and revision. The simulated no-AI condition represents an LLM response to instructions for completing a task without AI; it does not provide a human baseline. Classroom studies can test whether the task patterns observed here also emerge when students select sources, revise judgments, and defend their decisions, and whether these patterns extend to argument quality or subsequent learning.

Several scored features were directly elicited by the task instructions. Higher scores therefore describe how successfully a format prompted writers to state their use of evidence, treatment of objections, and reasons for revision. Their correspondence with students' actual decisions remains an empirical question. Linking submitted work with source choices, successive drafts, and follow-up defenses would clarify when written explanations track the decisions made during composition and when they primarily reflect the task's reporting requirements. The selected questionnaire stimuli and item wording may also have cued participants to attend to the targeted features.

Questionnaire responses distinguished broad task functions, while the five three-item category scores showed limited internal consistency, constraining fine-grained separation among individual features. Perceived-feature and assessment-diagnosticity ratings came from the same respondents, and participants were drawn from one university; shared response tendencies and course experience may have shaped the observed associations. Studies across disciplines, courses, and evaluator groups can examine whether similar argumentative cues guide judgments in different educational settings.

The open-ended responses identify the cues participants associated with thinking and AI-like writing, but the analysis is available at the level of coded themes. The retained dataset contains coded responses but not the original verbatim text, so illustrative quotations cannot be verified and individual responses cannot be examined in their original context. Future studies that retain full responses and link them to evaluator judgments and writing-process records could distinguish judgments grounded in argumentative quality from reactions to stylistic features associated with thinking or AI use.

## 8 Conclusion

Task structure changed the composition of the generated responses. Evidence-integration memoranda concentrated source selection and source–claim explanation, follow-up-and-revision defenses concentrated responses to challenge and reasons for changing a judgment, and position essays showed the greatest movement when responsible collaboration added disclosure, verification, and revision requirements. Participant ratings and open-ended responses followed the same division, linking informative assessment materials with evidence integration, comparison of reasons, and revision.

GenAI-mediated assessment therefore depends on the information a task requires writers to provide about their decisions. Assignments that specify source use, challenge, and revision give AI-use governance a substantive place within the response and make the resulting material more informative for evaluation. Controlled LLM simulation can pretest these design choices before deployment; classroom studies with authentic student work are the next step for examining their transfer.

## Data Availability

The raw data supporting the conclusions of this article will be made available by the authors, without undue reservation.
