# Supplementary material for: Making argumentative work visible in GenAI-mediated assessment: an exploratory LLM simulation and questionnaire study in university ideological and political education
Source: Front Psychol. 2026 Oct 30;17:1908308. doi: 10.3389/fpsyg.2026.1908308 (PMC13759825; doi:10.3389/fpsyg.2026.1908308)
Supplement: Supplementary file 1 [file Data_Sheet_1.pdf]

frontiers

Supplementary Material

1 LLM GENERATION PROTOCOL

Text generation was conducted in Chinese using DeepSeek-V3.1 through the official DeepSeek API and the `deepseek-chat` endpoint. Generation took place from 18 to 24 March 2026. Web browsing, external search, retrieval-augmented generation, and tool calling were disabled. Table S1 reports the generation parameters and session rules.

Table S1. LLM generation settings.

Field	Setting
Model family	DeepSeek-V3.1
API endpoint	`deepseek-chat` through the official DeepSeek API
Generation language	Chinese
Generation period	18–24 March 2026; China Standard Time (UTC+8)
Temperature	0.70
Top-p	0.90
Maximum output tokens	2200
Frequency penalty	0
Presence penalty	0
LLM sampling seed	Not set
Generation-order seed	20260318
Streaming	False
Web browsing and external search	Disabled
Retrieval-augmented generation (RAG)	Disabled
Tool calling	Disabled
Session design	Independent session for each candidate; follow-up turns remained within the corresponding defense session
Retry rule	Requests were repeated only after technical failure; outputs were not regenerated on the basis of content quality

Each candidate was generated in an independent API session. Conversation history, outputs from other candidates, and few-shot examples were not shared across sessions. The follow-up-and-revision defense retained its initial response, two standardized follow-up turns, and final revision within a single artifact-specific session. Technical failures were retried; content quality was addressed through the prespecified screening procedure described in Section 3. Request-level API logs and server-side revision identifiers were unavailable in the study record.

1.1 Shared Prompt Framework

The same system instruction was applied across all domains, tasks, and simulation scenarios. The English rendering below preserves the content of the Chinese generation prompt.

> You are participating in a text-simulation study of university course assessment. Generate a complete course-assignment text from the thematic domain, source package, task instruction, and simulation scenario supplied by the researcher. Use only the information in the supplied Source Materials. Do not use external sources or add unverifiable numbers, facts, policies, cases, authors, URLs, or citations. Compare different emphases in the materials when the task requires comparison, and do not erase those differences. Follow the current Task

> Instruction and Simulation Scenario exactly. Do not mention the research design, experimental conditions, scoring rubric, TRASI, PV, EA, SC, CA, or RR unless the current scenario explicitly requires disclosure of AI use. Do not introduce spelling errors or artificial "human-like" errors and do not discuss AI-detection tools. Write a complete university assignment rather than an explanation of how the prompt was executed. Ensure that every claim and item of evidence can be traced to the provided materials. If information is uncertain, do not invent it. Output only the requested final assignment text.

Each request followed the same user-prompt structure, with the domain, issue, source materials, task instruction, simulation scenario, and output fields filled for the assigned condition.

```
[THEMATIC DOMAIN]
{domain_name}

[ASSESSMENT ISSUE]
{issue}

[SOURCE MATERIALS]
Material 1
Title: {source_1_title}
Content: {source_1_content}

Material 2
Title: {source_2_title}
Content: {source_2_content}

These materials are the complete factual basis for the task.
Do not use any other source.

[TASK INSTRUCTION]
{task_prompt}

[SIMULATION SCENARIO]
{scenario_prompt}

[OUTPUT REQUIREMENTS]
Write in Chinese. Make the position clear and connect evidence to judgment.
Do not invent data, policies, people, events, or sources. Distinguish facts,
interpretation, and value judgment. Complete the specified word range and
output only the final task text.
```

2 EXPERIMENTAL CONDITIONS AND TASK INSTRUCTIONS

The simulation crossed three AI-involvement scenarios with three task formats. The scenario prompts varied how AI participation and the associated checking, disclosure, and revision processes appeared in the generated artifact.

The scenario blocks differed in the visibility of AI involvement and the required process account. They did not instruct the model to evade AI detection or to introduce deliberate errors.

Simulated no-AI scenario. Simulate a student who independently read the provided materials and independently formed the position, selected evidence, organized the argument, and wrote the text. The student did not use generative AI for planning, drafting, revision, or language editing. Do not mention AI or add a statement that AI was not used. Use only the supplied materials.

Responsible-collaboration scenario. Simulate a student who may use generative AI for issue organization, alternative arguments, structure, or expression, while retaining responsibility for the final judgment. The student must check important facts and claims against the supplied materials, compare AI suggestions with their own judgment, revise or reject unsupported or overgeneralized suggestions, disclose naturally which stages used AI, and give reasons for important changes. Do not claim external searches or use facts outside the supplied materials.

Concealed-ghostwriting scenario. Generate a complete submittable assignment directly with generative AI. Do not disclose AI participation, describe the generation process, or provide tool records. Use only the supplied materials and do not invent facts, sources, policies, data, URLs, or personal experiences. Do not attempt to evade AI detection or introduce deliberate human-like errors.

Table S2 summarizes the task instructions and output ranges. Position essays required a judgment and supporting reasons; evidence-integration memoranda required explicit source–claim links and source-selection rationales; follow-up-and-revision defenses added two standardized challenges and a final revised defense.

Table S2. Task instructions and output ranges.

Task type	Task instruction and output range
Position essay	State a clear position on the assessment issue and explain the main reasons using the source package. Target: 900–1100 Chinese characters.
Evidence-integration memorandum	Identify relevant evidence, explain how it supports each claim, and justify source selection. Target: 1000–1200 Chinese characters.
Follow-up-and-revision defense	Submit an initial response, complete the two standardized follow-up questions in the same session, and produce a final revised defense. Final body: 1000–1200 Chinese characters; revision rationale: 120–180 Chinese characters.

The two follow-up questions and final revision instruction were identical for all defense artifacts.

```
Follow-up Question 1
Re-examine your initial answer. From a perspective different from your
current position, state the strongest objection and assess whether the
initial
answer adequately addressed it. Explain the core of the objection, which
provided materials support or limit it, which parts of the original argument
remain valid, and what requires further support. Use only the current Source
Materials and add no external facts or sources.
```

```
Follow-up Question 2
After considering the preceding objection, reassess the original conclusion.
State which core judgments remain, which need modification, which require an
additional condition or scope limitation, and which materials support those
adjustments. Distinguish facts, interpretations, and value judgments. Do not
add information outside the Source Materials.

Final Revision
Using the initial answer and both follow-up rounds, submit a complete,
continuous revised text rather than a sequence of answers. State the final
position, retain necessary objections and responses, connect important
claims
to the supplied materials, and express any needed conditions or limitations
in the body. Add a section headed ``Revision rationale'' of 120--180 Chinese
characters explaining what was retained, changed, or qualified and why.
```

3 SOURCE PACKAGES AND TEXT GENERATION

Three fixed source packages were prepared, one for each thematic domain. All task and scenario conditions within a domain received the same materials, and generation was restricted to those sources. The packages and assessment issues are listed in Table S3.

Table S3. Thematic domains and fixed source packages.

Thematic domain	Source package and assessment issue
Public responsibility and rule consciousness	Personal Information Protection Law of the People's Republic of China, together with official Civil Code materials on personality rights, privacy, and personal-information protection. Issue: the boundary between personal rights and personal-information use in digital public life.
Social participation and value judgment	Regulations on Volunteer Service and the Charity Law of the People's Republic of China. Issue: whether university volunteer-service assessment should emphasize service hours or actual public contribution.
National narratives and analysis of real-world issues	*China's Green Development in the New Era* and the 2025 Statistical Communique of the People's Republic of China on National Economic and Social Development. Issue: the relationship among individual responsibility, public governance, and industrial structure in a green and low-carbon transition.

The complete design contained 27 domain–task–scenario cells. Seven candidates were generated in each cell, producing 189 initial artifacts. Screening preceded formal TRASI scoring and assigned one primary exclusion reason to each excluded artifact. Forty-one artifacts were excluded: 14 for an incomplete task response, 13 for duplication or high similarity, and 14 for insufficient evaluability. The final set comprised 148 artifacts. Tables S4–S7 report the criteria and retained allocation.

Table S4. Artifact screening criteria and retention.

Criterion	n	Definition
Failure of completeness	14	Output was truncated, did not complete the assigned task, or omitted a major required component.
Duplicate or highly similar output	13	Core argument, evidence organization, and paragraph structure substantially duplicated another artifact in the same condition.
Insufficient evaluability	14	Output was too short, consisted mainly of generic assertions, or lacked sufficient content for stable coding of the prespecified dimensions.
Total excluded	41	Each excluded artifact received one mutually exclusive primary reason.
Final retained sample	148	Retention rate: 78.3%.

Table S5: Domain-by-task-by-scenario candidate screening record.

Domain	Task	Scenario	Generated	Retained	INC	DUP	EVAL
D1	PE	NAI	7	4	1	1	1
D2	PE	NAI	7	5	1	1	0
D3	PE	NAI	7	5	1	0	1
D1	PE	RC	7	6	0	1	0
D2	PE	RC	7	6	0	0	1
D3	PE	RC	7	5	1	1	0
D1	PE	CG	7	4	1	1	1
D2	PE	CG	7	4	1	1	1
D3	PE	CG	7	4	1	1	1
D1	EM	NAI	7	7	0	0	0
D2	EM	NAI	7	7	0	0	0
D3	EM	NAI	7	6	0	0	1
D1	EM	RC	7	7	0	0	0
D2	EM	RC	7	6	1	0	0
D3	EM	RC	7	7	0	0	0
D1	EM	CG	7	6	0	1	0
D2	EM	CG	7	7	0	0	0
D3	EM	CG	7	6	0	0	1
D1	FRD	NAI	7	5	1	1	0
D2	FRD	NAI	7	6	0	0	1
D3	FRD	NAI	7	5	1	1	0
D1	FRD	RC	7	6	0	0	1
D2	FRD	RC	7	5	1	1	0
D3	FRD	RC	7	6	0	0	1
D1	FRD	CG	7	4	1	1	1
D2	FRD	CG	7	4	1	1	1
D3	FRD	CG	7	5	1	0	1

Domain	Task	Scenario	Generated	Retained	INC	DUP	EVAL
Total			189	148	14	13	14

INC = incomplete task response; DUP = duplicate or highly similar output; EVAL = insufficient evaluability.

Table S6. Final allocation by task and simulation scenario.

Task type	Simulated no-AI	Responsible collaboration	Concealed ghostwriting	Total
Position essay	14	17	12	43
Evidence-integration memorandum	20	20	19	59
Follow-up-and-revision defense	16	17	13	46
Total	50	54	44	148

Table S7. Retained artifacts by domain, task, and simulation scenario.

Task type	Simulated no-AI	Responsible collaboration	Concealed ghostwriting	Total
Position essay	14 (4/5/5)	17 (6/6/5)	12 (4/4/4)	43
Evidence-integration memorandum	20 (7/7/6)	20 (7/6/7)	19 (6/7/6)	59
Follow-up-and-revision defense	16 (5/6/5)	17 (6/5/6)	13 (4/4/5)	46
Total	50	54	44	148

4 TRASI CODING FRAMEWORK

The Textual Response Artifact Scoring Index (TRASI) was used as an artifact-level rubric for five observable properties of the generated texts. Process visibility (PV) coded whether the path from source interpretation and evidence selection to judgment formation was traceable in the artifact (Toulmin, 2003; Berland and Reiser, 2009). Evidence anchoring (EA) coded whether major claims were connected to identifiable supplied material and whether the evidential function of that material was explained (Toulmin, 2003; Berland and Reiser, 2009; Osborne, 2010). PV therefore concerned the visibility of the reasoning path, whereas EA focused on the quality of the source–claim connection.

Contextual embeddedness (SC) coded whether the argument addressed the actors, institutions, practical constraints, and conditions relevant to the assigned issue (Brown et al., 1989; Gulikers et al., 2004). Counterargument handling (CA) coded the identification, fair representation, and reasoned response to a substantive objection (Kuhn, 1991; Nussbaum and Schraw, 2007; Osborne, 2010). SC located the judgment within its practical setting; CA assessed how the text handled a competing position within that setting.

Revision-rationale clarity (RR) coded what was retained, changed, or qualified, which evidence or objection prompted reconsideration, and why the final judgment followed (Flower and Hayes, 1981; Nicol and Macfarlane-Dick, 2006; Chinn and Brewer, 1993). RR required an account of change and its justification, distinguishing it from general process description under PV. Scores referred to evidence visible in the artifact. Table S8 summarizes the five dimensions, and Table S9 provides the complete 1–5 anchors. The overall TRASI score was the arithmetic mean of the five dimension scores.

Table S9: Anchored 1–5 TRASI scoring rubric.

Dimension	Score	Anchor
PV	1	Presents only a conclusion or generic reason; the path from material to judgment is not identifiable.
PV	2	Uses general process language such as “according to the materials” or “after considering,” but does not specify evidence selection, comparison, or judgment steps.

Supplementary Table S9 continued

Dimension	Score	Anchor
PV	3	Shows part of a material–reason–conclusion relationship, but the reasoning chain, evidence selection, comparison, or weighting remains incomplete.
PV	4	Clearly presents the main steps of interpreting material, selecting or comparing evidence, and forming the judgment; the central reasoning path is traceable.
PV	5	Systematically links material identification, evidence comparison, decision criteria, weighting, qualification, and final conclusion in a continuous and highly traceable chain.
EA	1	Core claims lack explicit evidence or no identifiable link connects the material to the claim.
EA	2	Mentions a source or policy only generally and does not explain how the material supports the judgment.
EA	3	Connects some core claims to specific material, but explains evidential function incompletely or leaves important claims unsupported.
EA	4	Anchors most core judgments in identifiable material and explains the supporting relationship between evidence and claim.
EA	5	Provides a systematic claim–evidence–explanation structure, distinguishes fact, interpretation, and value judgment, and addresses the scope and limits of the evidence.
SC	1	Remains at the level of abstract values or principles and provides no concrete issue context.
SC	2	Mentions a real-world issue but gives little analysis of actors, institutional setting, or implementation conditions.
SC	3	Identifies at least one material contextual factor, such as an actor, institutional requirement, resource condition, or practical limitation, but connects it only weakly to the conclusion.
SC	4	Integrates multiple contextual factors and adjusts the judgment in relation to concrete institutional, actor, or practical conditions.
SC	5	Sustains attention to the concrete issue environment, actors, institutions, resources, responsibility boundaries, and implementation constraints, and states the conditions and scope under which the conclusion applies.
CA	1	Contains no counterposition, competing explanation, or substantive objection.
CA	2	Notes that other views exist but does not state their reasons or answer them directly.
CA	3	States at least one substantive counterargument and offers a basic response, but the response is simple or insufficiently supported.
CA	4	Represents a challenging counterargument substantially and responds directly with supplied material or reasoning.
CA	5	Handles a strong counterargument accurately, acknowledges its reasonable part, explains its boundary of application, and uses it to strengthen, qualify, or modify the original judgment.
RR	1	Presents no change in judgment, revision process, or reason for revision.
RR	2	States that the response was reconsidered or revised without specifying what changed or why.
RR	3	Identifies at least one specific change and gives a general reason for it.
RR	4	Clearly states the original judgment, revised judgment, and the specific evidence, objection, or logical problem that prompted the change.
RR	5	Presents a complete original judgment–trigger–re-evaluation–retention/modification/qualification–final judgment chain and explains why each component was retained or changed.

4.1 Top-Anchor Scoring Examples

The following excerpts illustrate the textual evidence associated with a score of 5 on each dimension.

PV, score 5. Material 1 emphasizes that processing personal information should have a specified purpose, while Material 2 shows that personality rights cannot be compressed without limit for technical convenience. Because the materials address different levels, efficiency alone cannot determine whether collection is reasonable. Necessity is considered first, followed by whether the collection scope is directly related to the service; convenience may be relevant but cannot justify unlimited collection.

EA, score 5. Material 1 states that personal-information processing should have an explicit and reasonable purpose. This does not support a prohibition on data use; it supports a necessary relationship between the processing scope and the stated purpose. If the same service can operate with less information, expanded collection requires an additional justification.

Table S8. TRASI dimensions and coding focus.

Code	Operational focus
PV: process visibility	Whether the text makes steps of source use, decision making, checking, or revision inspectable.
EA: evidence anchoring	Whether claims are tied to identifiable supplied evidence and the evidential function is explained.
SC: contextual embeddedness	Whether the argument connects the supplied issue to a concrete institutional, social, or course context rather than relying on generic statements.
CA: counterargument handling	Whether a plausible objection is identified, represented fairly, and answered with material-based reasoning.
RR: revision-rationale clarity	Whether changes in position, organization, or wording are explained by reference to evidence, objections, or scope conditions.

SC, score 5. Volunteer-service hours record participation, but long-term community support, one-time events, and professional services do not have a fixed relationship between time and public benefit. In a university management context, hours can function as a basic record, but using them as the main criterion may ignore task type, recipient needs, and actual contribution.

CA, score 5. A counterargument is that service hours are more objective and easier to administer uniformly. This is reasonable with respect to measurement cost, but ease of measurement does not show that hours fully represent public contribution. Hours should therefore remain a basic record while service content, difficulty, recipient feedback, and completion quality are also considered.

RR, score 5. The initial response treated individual low-carbon behavior as the principal source of responsibility. After comparing the materials again, the role of individual behavior was retained, but it was no longer described as the primary determining factor. Because the materials also address public governance and industrial structure, the final judgment states that individual action matters but its effect depends on infrastructure, industrial conditions, and policy.

5 HUMAN RATING AND INTER-RATER RELIABILITY

Two human raters independently evaluated all 148 artifacts; no automated or LLM-assisted scoring was used. One rater had doctoral training in ideological and political education or higher education and approximately six years of experience in university teaching, course assessment, or evaluation of argumentative assignments. The second had doctoral training in educational psychology, educational evaluation, or higher education and approximately five years of experience in textual coding, educational measurement, argument analysis, or rubric-based assessment. A third senior researcher with relevant educational-evaluation experience served as adjudicator.

The raters completed approximately two hours of rubric training and two calibration rounds using 20 pilot artifacts outside the analytical sample. The first round used 12 artifacts to clarify distinctions among PV, EA, SC, CA, and RR; the second used eight additional artifacts to confirm the scoring rules. Formal artifacts were relabeled T001–T148, task and scenario labels were removed, and each rater received a separately randomized order. Raters worked independently and did not access each other's scores before the initial rating database was locked. They were blind to the assigned labels, although AI disclosure or revision structure could remain visible in the artifact.

Agreement was calculated from the independent ratings before reconciliation. Quadratic-weighted Cohen's κ was estimated separately for PV, EA, SC, CA, and RR, with 2,000 artifact-level bootstrap resamples for confidence intervals. Overall-score agreement used two-way random-effects, absolute-agreement ICC(2,1); ICC(2,2) describes agreement for the mean of the two raters. One-point differences were resolved through rubric-based discussion. Differences of two or more points, or cases unresolved after discussion, were referred to the third adjudicator. Substantive analyses used the reconciled ratings. Table S10 reports the agreement coefficients.

6 QUESTIONNAIRE PARTICIPANTS AND PROCEDURE

The questionnaire was administered through Wenjuanxing from 2 to 20 April 2026. Participants were recruited voluntarily through Central South University course groups, year-level groups, graduate-student discussion groups, and faculty work groups. No paid sample service or commercial survey panel was used. Eligible participants were at least 18 years old, affiliated with the university, able to read the Chinese materials, willing to provide electronic informed consent, and able to complete the assigned material evaluation.

Table S10. TRASI inter-rater reliability from unreconciled ratings.

Dimension/statistic	Estimate	95% CI
PV: quadratic-weighted Cohen's κ	.82	[.76, .87]
EA: quadratic-weighted Cohen's κ	.79	[.72, .85]
SC: quadratic-weighted Cohen's κ	.76	[.69, .82]
CA: quadratic-weighted Cohen's κ	.81	[.75, .86]
RR: quadratic-weighted Cohen's κ	.84	[.79, .89]
Overall TRASI: ICC(2,1)	.89	[.85, .92]
Overall TRASI: ICC(2,2)	.94	[.92, .96]

ICC(2,1) is the two-way random-effects, absolute-agreement, single-measure coefficient; ICC(2,2) is the corresponding coefficient for the mean of two raters. Confidence intervals for the weighted κ values used 2,000 artifact-level bootstrap resamples.

Of 289 submitted records, 8 incomplete questionnaires, 7 records that failed at least one instructed-response attention check, and 6 records completed in under 6 minutes were excluded before analysis. The final sample contained 268 participants and complete data for the required quantitative items; no imputation was applied. Participants received no cash, course credit, gifts, or other material incentives. Participant composition, screening, relevant course experience, and GenAI-use frequency are reported in Tables S11–S14.

All participants read an electronic information and consent page before entering the questionnaire. It described the study purpose and procedure, expected duration, voluntary participation, withdrawal before submission, anonymous analysis, aggregate reporting, and evaluation of AI-generated course materials. Participation required confirmation of age and consent. Under Central South University requirements for anonymous, minimum-risk educational surveys, formal ethics-committee review was not required, and no separate approval number was issued. No directly identifying or sensitive personal information was collected. The analytical file used anonymous response IDs and non-identifying study variables.

Table S11. Questionnaire participant composition.

Participant category	n	%
Undergraduate students	174	64.9
Graduate students	49	18.3
University teachers	37	13.8
Other university-affiliated personnel	8	3.0
Total	268	100.0

Table S12. Questionnaire record screening flow.

Screening step	Records excluded	Records remaining
Initial questionnaire records	–	289
Incomplete questionnaire	8	281
Failed at least one attention check	7	274
Completion time under 6 minutes	6	268
Total exclusions	21	–
Final valid sample	–	268 (92.7% of initial records)

The multiple-response GenAI item asked about information search or concept explanation, learning support, brainstorming, writing outlines, text drafting, language polishing, translation, programming or data analysis, non-use, and other uses. Percentages may exceed 100% because respondents could select more than one activity.

Table S13. Prior course experience and GenAI use among questionnaire respondents.

Background variable and category	n	%
Ideological and political theory course experience		
Completed at least one course	247	92.2
Currently taking a course	16	6.0
Never taken a course	5	1.9
Completed or currently taking at least one	263	98.1
GenAI use frequency in the preceding six months		
Never	28	10.4
Rarely (less than once per month on average)	47	17.5
Monthly (at least monthly, less than weekly)	62	23.1
Weekly (at least weekly, not almost daily)	91	34.0
Daily or almost daily	40	14.9
Three-level descriptive grouping of GenAI experience		
Low/no experience (never + rarely)	75	–
Moderate experience (monthly)	62	–
Frequent experience (weekly + daily/almost daily)	131	–

Table S14. Self-reported GenAI uses during the preceding six months.

Activity	n	% of total sample	% of ever-users
Information search or concept explanation	188	70.1	78.3
Learning support	176	65.7	73.3
Brainstorming	142	53.0	59.2
Writing outlines	126	47.0	52.5
Text drafting	119	44.4	49.6
Language polishing	153	57.1	63.8
Translation	137	51.1	57.1
Programming or data analysis	89	33.2	37.1
Never used	28	10.4	–
Other	21	7.8	8.8

The denominator for "% of ever-users" was 240 (268 total respondents minus 28 who reported never using GenAI). The "never used" and "other" options were retained as displayed response categories.

7 STIMULUS ALLOCATION AND MANIPULATION CHECKS

The questionnaire crossed the three task formats with the three simulation scenarios. The nine cells contained 29 or 30 participants, and each participant evaluated one artifact. Three different artifacts were used in each cell, one from each thematic domain, yielding 27 questionnaire stimuli. In cells with 30 participants, each artifact was evaluated by 10 participants; in cells with 29 participants, the allocation was 10, 10, and 9.

Stimulus selection was completed before formal TRASI scoring. Eligible artifacts were complete, non-duplicative, untruncated, consistent with the assigned task and scenario, based on the supplied sources, free of obvious factual hallucinations, within the specified length range, and suitable for participant evaluation. Table S16 lists the 27 final assignments. The contemporaneous randomization log is unavailable. Participants saw the task instructions, assigned artifact, and necessary background information. Formal condition labels were not displayed; responsible-collaboration artifacts could disclose AI use as part of the text.

Table S15. Questionnaire task–scenario allocation and stimulus selection.

Task type	Simulated no-AI	Responsible collaboration	Concealed ghostwriting	Total
Position essay	30	30	30	90
Evidence-integration memorandum	29	30	29	88
Follow-up-and-revision defense	30	30	30	90
Total	89	90	89	268

Each task–scenario cell contained three artifacts, one from each thematic domain. Cells with 30 respondents used a 10/10/10 evaluator distribution; cells with 29 respondents used 10/10/9. Stimulus selection preceded formal TRASI scoring.

Table S16: Questionnaire stimulus assignment.

Domain	Task	Scenario	Selected retained artifact
D1	PE	NAI	D1_PE_NAI_R04
D2	PE	NAI	D2_PE_NAI_R03
D3	PE	NAI	D3_PE_NAI_R01
D1	PE	RC	D1_PE_RC_R06
D2	PE	RC	D2_PE_RC_R01
D3	PE	RC	D3_PE_RC_R02
D1	PE	CG	D1_PE_CG_R02
D2	PE	CG	D2_PE_CG_R01
D3	PE	CG	D3_PE_CG_R01
D1	EM	NAI	D1_EM_NAI_R01
D2	EM	NAI	D2_EM_NAI_R05
D3	EM	NAI	D3_EM_NAI_R05
D1	EM	RC	D1_EM_RC_R07
D2	EM	RC	D2_EM_RC_R05
D3	EM	RC	D3_EM_RC_R03
D1	EM	CG	D1_EM_CG_R02
D2	EM	CG	D2_EM_CG_R02
D3	EM	CG	D3_EM_CG_R06
D1	FRD	NAI	D1_FRD_NAI_R04
D2	FRD	NAI	D2_FRD_NAI_R05
D3	FRD	NAI	D3_FRD_NAI_R04
D1	FRD	RC	D1_FRD_RC_R05
D2	FRD	RC	D2_FRD_RC_R04
D3	FRD	RC	D3_FRD_RC_R04
D1	FRD	CG	D1_FRD_CG_R02
D2	FRD	CG	D2_FRD_CG_R01
D3	FRD	CG	D3_FRD_CG_R04

R01, R02, and subsequent identifiers denote the retained-artifact numbering within the corresponding domain–task–scenario cell.

Six five-point items assessed recognition of the task and scenario features. B1–B3 addressed source integration, evidence-selection rationale, and response-and-revision structure; B4–B6 addressed AI disclosure, verification, and the treatment of AI suggestions. Table S17 provides the item wording, and Table S18 reports the condition-level results.

Table S17. Section B manipulation-check and material-comprehension items.

Item	Wording	Primary target
B1	The material requires or shows integration of information from multiple sources rather than expression of a final position alone.	Evidence-integration task
B2	The material shows why particular evidence was selected and used to support a specific judgment.	Evidence-integration task
B3	The material responds to further questioning or opposing views and shows reconsideration or revision of the original judgment.	Follow-up-and-revision task
B4	The material explicitly indicates that generative AI participated in completing the assignment.	Responsible collaboration
B5	The material shows a process of checking information or suggestions provided by AI.	Responsible collaboration
B6	The material explains which AI suggestions were accepted, modified, or rejected and why.	Responsible collaboration

All items used a five-point scale: 1 = strongly disagree, 2 = disagree, 3 = neither agree nor disagree, 4 = agree, and 5 = strongly agree.

Table S18. Manipulation-check results.

Item	Grouping	Means (ordered groups)	Overall M	Overall SD	$F(2, 265)$	Partial η^2
B1	Task type	2.41 (.82) / 4.25 (.61) / 3.44 (.76)	3.36	1.05	139.77	.513
B2	Task type	2.28 (.78) / 4.18 (.65) / 3.36 (.79)	3.27	1.07	146.39	.525
B3	Task type	1.96 (.72) / 2.35 (.79) / 4.41 (.58)	2.91	1.29	316.40	.705
B4	Scenario	1.52 (.59) / 4.46 (.55) / 1.43 (.52)	2.48	1.52	868.09	.868
B5	Scenario	1.78 (.68) / 4.28 (.61) / 1.72 (.64)	2.60	1.36	461.69	.777
B6	Scenario	1.64 (.62) / 4.16 (.66) / 1.69 (.63)	2.50	1.34	458.68	.776

Values in the ordered-groups column are M (SD). For B1–B3, the groups are position essay, evidence-integration memorandum, and follow-up-and-revision defense. For B4–B6, they are simulated no-AI, responsible collaboration, and concealed ghostwriting. All omnibus tests had $p < .001$.

Nine participants (3.4%) met the prespecified nonpassing criterion, defined as responses opposite to the assigned manipulation on at least two relevant check items. They remained in the primary analysis. Excluding them produced $N = 259$, preserved the ordering of task and scenario means, and yielded effect estimates in the same substantive range.

8 OPEN-ENDED RESPONSE CODING

Four open-ended questions asked participants to identify textual evidence of thinking (H1), formulaic or AI-like cues (H2), useful additions to assessment tasks (H3), and priorities for assessment reform (H4). The coding unit was the smallest meaningful proposition within a response. Multi-label coding allowed one response to receive several themes; ambiguous material was coded only when its meaning could be supported by the text.

Two trained coders independently coded all usable responses after approximately two hours of dictionary training and calibration on 30 pilot responses excluded from formal reliability analysis. Theme-level Cohen's κ was calculated from the independent present/absent decisions before reconciliation. Disagreements were discussed with reference to the coding dictionary, and unresolved cases were adjudicated by a third educational researcher. Tables S19–S23 provide the question structure, decision rules, theme frequencies, and agreement coefficients. The retained qualitative dataset contains the coded open-ended responses but not the original verbatim text; consequently, illustrative quotations cannot be independently verified or reported from the retained archive.

Table S19. Open-ended questions and thematic code families.

Item	Themes
H1: features that appeared to reflect a thinking process	Evidence integration; comparative reasoning; revision rationale; contextual embeddedness; counterargument response.
H2: formulaic expression or apparent AI traces	Formulaic structure; over-polished expression; unsupported conclusion; generic abstraction; repetitive pattern; vague attribution.
H3: requirements that might reduce misjudgment	Evidence table; draft/revision trajectory; AI-use disclosure; follow-up mechanism; rationale for source selection.
H4: possible directions for assessment design	Process-oriented task design; scoring-criteria reform; AI-use rules; follow-up/defense; multi-stage submission.

Table S20. H1 and H2 multi-label coding dictionaries.

Code	Theme	Decision rule
H1-EI	Evidence integration	Mentions integrating, connecting, or synthesizing multiple sources, materials, or pieces of evidence.
H1-CR	Comparative reasoning	Mentions comparing different views, evidence, criteria, or explanations.
H1-RR	Revision rationale	Mentions a changed judgment and why the response was modified or retained.
H1-CE	Contextual embeddedness	Emphasizes analysis of concrete actors, policies, institutions, situations, or practical constraints.
H1-CA	Counterargument response	Mentions an opposing view, objection, or disagreement and a response to it.
H2-FM	Formulaic structure	Mentions a fixed template, routine structure, or mechanical first–second–finally pattern.
H2-OP	Over-polished expression	Mentions language that is unusually complete, even, fluent, or lacking individual variation.
H2-UC	Unsupported conclusion	Mentions a clear conclusion lacking material, evidence, or argumentative support.
H2-GE	Generic abstraction	Mentions vague, broad, or principle-only content without a concrete context.
H2-RP	Repetitive pattern	Mentions repeated wording, sentence forms, or paragraph structures.
H2-VA	Vague attribution	Mentions unclear attributions such as research shows or the materials indicate without a source.

Only explicit textual evidence was coded. General praise without a supporting reason was not assigned one of these codes.

Table S21. H3 and H4 multi-label coding dictionaries.

Code	Theme	Decision rule
H3-ET	Evidence table	Suggests an evidence table, source table, or material–claim correspondence table.
H3-DR	Draft/revision trajectory	Suggests retaining an initial draft, revised draft, version history, or revision trail.
H3-AI	AI-use disclosure	Suggests disclosing AI use, stages of use, or scope of use.
H3-FU	Follow-up mechanism	Suggests teacher questioning, oral defense, random follow-up, or a second explanation.
H3-RS	Rationale for source selection	Requires an explanation of why a source or item of evidence was selected.
H4-PT	Process-oriented task design	Suggests replacing a one-off product with a process or multi-stage task.
H4-SC	Scoring-criteria reform	Suggests increasing the weight of evidence, reasoning, or revision indicators.
H4-AR	AI-use rules	Suggests explicit rules for permitted AI use, disclosure, and responsibility.
H4-FD	Follow-up/defense	Suggests a defense, follow-up, oral explanation, or in-person clarification.
H4-MS	Multi-stage submission	Suggests staged submission of an outline, materials, draft, revision, and final version.

Table S22. Open-ended theme frequencies and denominators.

Question	Theme	n	% of valid texts
H1 ($n = 262$)	Evidence integration	158	60.3
	Comparative reasoning	131	50.0
	Revision rationale	116	44.3
	Contextual embeddedness	101	38.5
	Counterargument response	94	35.9
H2 ($n = 258$)	Formulaic structure	146	56.6
	Over-polished expression	118	45.7
	Unsupported conclusion	103	39.9
	Generic abstraction	91	35.3
	Repetitive pattern	82	31.8
	Vague attribution	74	28.7
H3 ($n = 261$)	Follow-up mechanism	149	57.1
	Draft/revision trajectory	137	52.5
	Evidence table	121	46.4
	Rationale for source selection	112	42.9
	AI-use disclosure	96	36.8
H4 ($n = 256$)	Process-oriented task design	151	59.0
	Scoring-criteria reform	133	52.0
	AI-use rules	111	43.4
	Follow-up/defense	98	38.3
	Multi-stage submission	87	34.0

Responses could receive more than one theme, so percentages do not sum to 100%. Percentages use the number of usable responses to each question as the denominator.

Table S23: Open-ended theme-level inter-coder agreement.

Question/code	Theme	Cohen's κ	95% CI
H1-EI	Evidence integration	.89	[.83,.95]
H1-CR	Comparative reasoning	.85	[.78,.92]
H1-RR	Revision rationale	.84	[.77,.91]
H1-CE	Contextual embeddedness	.81	[.73,.89]
H1-CA	Counterargument response	.79	[.70,.88]
H2-FM	Formulaic structure	.86	[.79,.93]
H2-OP	Over-polished expression	.83	[.75,.91]
H2-UC	Unsupported conclusion	.82	[.74,.90]
H2-GE	Generic abstraction	.80	[.71,.89]
H2-RP	Repetitive pattern	.79	[.70,.88]
H2-VA	Vague attribution	.76	[.66,.86]
H3-ET	Evidence table	.88	[.82,.94]
H3-DR	Draft/revision trajectory	.90	[.85,.95]
H3-AI	AI-use disclosure	.84	[.77,.91]
H3-FU	Follow-up mechanism	.87	[.81,.93]
H3-RS	Rationale for source selection	.82	[.74,.90]
H4-PT	Process-oriented task design	.88	[.82,.94]
H4-SC	Scoring-criteria reform	.85	[.78,.92]
H4-AR	AI-use rules	.83	[.75,.91]
H4-FD	Follow-up/defense	.81	[.72,.90]
H4-MS	Multi-stage submission	.78	[.68,.88]
H1 mean		.84	–
H2 mean		.81	–
H3 mean		.86	–
H4 mean		.83	–

Each coefficient was calculated from the independent present/absent coding decisions before discussion or adjudication. Confidence intervals are reported for the theme-level coefficients.

9 ADDITIONAL STATISTICAL RESULTS

Table S24 consolidates the prespecified effect sizes, confidence intervals, bootstrap settings, raw distribution underlying the largest questionnaire contrast, and correlations among the five TRASI categories. Table S25 reports the regression coefficients, collinearity statistics, residual checks, and influence diagnostics for perceived assessment diagnosticity.

Table S24. Effect sizes, confidence intervals, and bootstrap specifications.

Analysis	Contrast or factor	Estimate	95% CI	p
Simulation	Simulated no-AI − responsible collaboration	$d = -1.13$	$[-1.54, -0.72]$	$< .001$
Simulation	Responsible collaboration − concealed ghostwriting	$d = .88$	$[.46, 1.30]$	$< .001$
Simulation	Simulated no-AI − concealed ghostwriting	$d = -.39$	$[-.80, .02]$	.054
Simulation	Scenario ordinary η^2	.192	[.095,.286]	–
Simulation	Task ordinary η^2	.131	[.051,.224]	–
Simulation	Scenario × task ordinary η^2	.058	[.010,.128]	–
Questionnaire	Evidence memorandum − position essay	$d = 1.18$	[.86,1.50]	$< .001$
Questionnaire	Follow-up defense − position essay	$d = 1.37$	[1.05,1.69]	$< .001$
Questionnaire	Responsible collaboration − simulated no-AI	$d = 2.63$	[2.23,3.03]	$< .001$
Questionnaire	Responsible collaboration − concealed ghostwriting	$d = 1.58$	[1.24,1.92]	$< .001$
Questionnaire	Concealed ghostwriting − simulated no-AI	$d = .89$	[.58,1.20]	$< .001$
Questionnaire	Perceived TRASI: task partial η^2	.572	[.498,.632]	–
Questionnaire	Perceived TRASI: scenario partial η^2	.722	[.670,.763]	–
Questionnaire	Perceived TRASI: task × scenario partial η^2	.019	[.000,.051]	–
Questionnaire	Diagnosticity: task partial η^2	.266	[.178,.349]	–
Questionnaire	Diagnosticity: scenario partial η^2	.388	[.299,.465]	–
Questionnaire	Support for reform: task partial η^2	.423	[.335,.498]	–
Questionnaire	Support for reform: scenario partial η^2	.244	[.157,.326]	–
Questionnaire	AI transparency: scenario partial η^2	.036	[.002,.086]	–

Simulation ordinary eta-squared intervals used 5,000 bias-corrected and accelerated bootstrap resamples with the simulated artifact as the resampling unit. Figure 4 Cohen's d intervals used 5,000 bias-corrected and accelerated (BCa) bootstrap resamples, seed 20260324, the individual simulated artifact as the resampling unit, and each group retaining its original sample size. Questionnaire d intervals use the prespecified pairwise comparisons.

The raw perceived-TRASI distributions underlying the questionnaire contrast of $d = 2.63$ were: simulated no-AI, $n = 89$, $M = 2.70$, $SD = .22$, median 2.69, IQR 2.55–2.84, range 2.20–3.27; responsible collaboration, $n = 90$, $M = 3.29$, $SD = .23$, median 3.30, IQR 3.14–3.44, range 2.73–3.87; and concealed ghostwriting, $n = 89$, $M = 2.91$, $SD = .25$, median 2.91, IQR 2.74–3.08, range 2.27–3.53. The first two groups differed by .59 and had a pooled standard deviation of approximately .224.

Simulation dimension correlations (95% CI, Fisher z) were PV–EA .46 [.32,.58], PV–SC .74 [.66,.81], PV–CA .81 [.75,.86], PV–RR .71 [.62,.78], EA–SC .62 [.51,.71], EA–CA .41 [.27,.54], EA–RR .66 [.56,.74], SC–CA .57 [.45,.67], SC–RR .86 [.81,.90], and CA–RR .59 [.47,.69]. Questionnaire correlations with perceived assessment diagnosticity were PV .59 [.51,.66], EA .54 [.45,.62], SC .49 [.39,.58], CA .62 [.54,.69], and RR .58 [.49,.65], all $p < .001$.

Table S25. Regression coefficients and diagnostics.

Predictor	B	SE	Standardized β	95% CI	p	VIF	Tolerance
Intercept	.72	.16	–	[.41,1.04]	$< .001$	–	–
PV	.21	.05	.22	[.11,.31]	$< .001$	1.82	.55
EA	.18	.04	.19	[.10,.26]	$< .001$	1.44	.69
SC	.09	.05	.09	[-.01,.19]	.073	2.12	.47
CA	.24	.05	.26	[.14,.34]	$< .001$	1.71	.58
RR	.17	.05	.18	[.07,.27]	.001	2.08	.48

Model or diagnostic	Result
Sample and model fit	$N = 268$; $R^2 = .580$; adjusted $R^2 = .572$; $F(5, 262) = 72.38$, $p < .001$
VIF and tolerance	VIF = 1.44–2.12; tolerance = .47–.69
Residual normality	Shapiro–Wilk $W = .993$, $p = .25$
Homoscedasticity	Breusch–Pagan $\chi^2(5) = 7.84$, $p = .165$
Outlying residual	Maximum absolute studentized residual = 2.91; no value exceeded $\|3.0\|$
Influence	Maximum Cook's $D = .031$; no value approached 1

REFERENCES

Berland, L. K. and Reiser, B. J. (2009). Making sense of argumentation and explanation. *Science Education 93, 26–55* doi:10.1002/sce.20286

Brown, J. S., Collins, A., and Duguid, P. (1989). Situated cognition and the culture of learning. *Educational Researcher 18, 32–42* doi:10.3102/0013189X018001032

Chinn, C. A. and Brewer, W. F. (1993). The role of anomalous data in knowledge acquisition: A theoretical framework and implications for science instruction. *Review of Educational Research 63, 1–49* doi:10.3102/00346543063001001

Flower, L. and Hayes, J. R. (1981). A cognitive process theory of writing. *College Composition and Communication 32, 365–387* doi:10.2307/356600

Gulikers, J. T. M., Bastiaens, T. J., and Kirschner, P. A. (2004). A five-dimensional framework for authentic assessment. *Educational Technology Research and Development 52, 67–86* doi:10.1007/BF02504676

Kuhn, D. (1991). The skills of argument

Nicol, D. J. and Macfarlane-Dick, D. (2006). Formative assessment and self-regulated learning: a model and seven principles of good feedback practice. *Studies in Higher Education 31, 199– 218* doi:10.1080/03075070600572090

Nussbaum, E. M. and Schraw, G. (2007). Promoting argument-counterargument integration in students' writing. *The Journal of Experimental Education 76, 59–92* doi:10.3200/JEXE.76.1.59-92

Osborne, J. (2010). Arguing to learn in science: The role of collaborative, critical discourse. *Science 328, 463–466* doi:10.1126/science.1183944

Toulmin, S. E. (2003). The uses of argument. *, 2nd Edn. Cambridge: Cambridge University Press* doi:10.1017/CBO9780511840005
